# The cumulation of ill health and low agency in socially excluded city dwellers in the Netherlands: how to better identify high-risk/high-need population segments with public health survey data

**DOI:** 10.1186/s12939-021-01471-w

**Published:** 2021-07-19

**Authors:** Addi P. L. van Bergen, Annelies van Loon, Stella J. M. Hoff, Judith R. L. M. Wolf, Albert M. van Hemert

**Affiliations:** 1 GGD Hollands Midden, Parmentierweg 49, 2316ZV Leiden, The Netherlands; 2 Department of Psychiatry, Leiden University Medical Centre, Albinusdreef 2, 2333 ZA Leiden, The Netherlands; 3 GGD Amsterdam, Nieuwe Achtergracht 100, 1018 WT Amsterdam, The Netherlands; 4 The Netherlands Institute of Social Research|SCP, Bezuidenhoutseweg 30, 2594 AV Den Haag, The Netherlands; 5 Impuls, The Netherlands Center for Social Care Research, Radboud University Medical Center, Geert Grooteplein Zuid 21, 6525 GA Nijmegen, The Netherlands

**Keywords:** Social exclusion, Social determinants of health, Anxiety and depression, Personal control, Kessler-10, Pearlin mastery scale, Public health monitoring, Population health

## Abstract

**Background:**

Population segmentation and risk stratification are important strategies for allocating resources in public health, health care and social care. Social exclusion, which is defined as the cumulation of disadvantages in social, economic, cultural and political domains, is associated with an increased risk of health problems, low agency, and as a consequence, a higher need for health and social care. The aim of this study is to test social exclusion against traditional social stratifiers to identify high-risk/high-need population segments.

**Methods:**

We used data from 33,285 adults from the 2016 Public Health Monitor of four major cities in the Netherlands. To identify at-risk populations for cardiovascular risk, cancer, low self-rated health, anxiety and depression symptoms, and low personal control, we compared relative risks (RR) and population attributable fractions (PAF) for social exclusion, which was measured with the Social Exclusion Index for Health Surveys (SEI-HS), and four traditional social stratifiers, namely, education, income, labour market position and migration background.

**Results:**

The analyses showed significant associations of social exclusion with all the health indicators and personal control. Particular strong RRs were found for anxiety and depression symptoms (7.95) and low personal control (6.36), with corresponding PAFs of 42 and 35%, respectively. Social exclusion was significantly better at identifying population segments with high anxiety and depression symptoms and low personal control than were the four traditional stratifiers, while the two approaches were similar at identifying other health problems. The combination of social exclusion with a low labour market position (19.5% of the adult population) captured 67% of the prevalence of anxiety and depression symptoms and 60% of the prevalence of low personal control, as well as substantial proportions of the other health indicators.

**Conclusions:**

This study shows that the SEI-HS is a powerful tool for identifying high-risk/high-need population segments in which not only ill health is concentrated, as is the case with traditional social stratifiers, but also a high prevalence of anxiety and depression symptoms and low personal control are present, in addition to an accumulation of social problems. These findings have implications for health care practice, public health and social interventions in large cities.

**Supplementary Information:**

The online version contains supplementary material available at 10.1186/s12939-021-01471-w.

## Introduction

Changes in society and demographic trends are putting pressure on our health care system [1]. The ageing population is leading to an increase in multiple morbidities [2], while improved medical treatment is expanding the lifespan of individuals with these health conditions [3]. Over the coming years, health care expenditures in the Netherlands are expected to grow twice as fast as the economy [3]. Households in which social and medical problems accumulate bring in numerous professionals—often too late—and this puts pressure on municipal finances [3]. It is therefore more important than ever to deploy resources in health care, public health and social care in such a manner that the greatest health gains can be made. To help understand the needs of the population so that governances and services can be better planned and delivered, population segmentation and risk stratification are essential steps.

In Western countries, a strong socioeconomic gradient in health has been observed. Health appears to progressively increase with socioeconomic position [4] and to decrease with higher societal inequality [5]. Traditionally, education, income and profession are used as indicators for socioeconomic status [6], but other social stratifiers have also been used. The World Health Organization summarises the 8 stratifiers that are the most frequently assessed in health inequality monitoring, namely, place of residence (rural, urban, etc.), race or ethnicity, occupation, gender, religion, education, socioeconomic status and social capital or resources (PROGRESS) [7]. We expected that social exclusion (SE) would also be a good or even better candidate than these traditional social factors to describe and analyse the social stratification of health.

According to the World Health Organization (WHO), SE is rooted in an interplay of dynamic processes at the individual, household, community, country, and global levels. These processes are driven by unequal power relationships and lead to a cumulation of deprivations in the economic, social, cultural and political domains [8, 9]. There is ample evidence that SE impacts health and that, vice versa, ill health exacerbates social exclusion [10]. Mediation and moderation effects may also be in place [11–13]. In fact, health is so intricately linked to SE that it is considered by some as part and parcel of the concept itself [14].

In this paper, we explored SE as a promising stratifier for both health and low agency. Agency refers herein to the human capability to influence one’s functioning and the course of events by one’s actions [15]. According to Link and Phelan [16], differential access to resources such as knowledge, money, power, prestige and beneficial social connections is an important, or even the most important, reason why interventions to improve health are consistently less effective in low versus high socioeconomic groups. So-called “high agent” interventions do not work for low socioeconomic groups because participants must use their personal resources or “agency” to benefit [17–19]. Population interventions that require individuals to use a low level of agency, for example, food manufacturers reducing the salt content of bread, smoke-free public places and so-called “nudge” interventions, are likely to be most effective and equitable [17]. At the core of SE lies the inability of persons to participate fully in society and make full use of the benefits that society offers. SE reinforces feelings of powerlessness, alienation, demoralization and lack of self-esteem [20, 21]. We therefore expected that SE may also be a good candidate to describe and analyse the social stratification of low agency.

To validly measure SE in routine public health monitoring, we previously developed the Social Exclusion Index for Health Surveys (SEI-HS) [22]. The measurement of SE in public health research is still in its infancy, and a generally accepted valid measure has not yet been developed [9]. Limitations related to earlier measures include a limited focus on only one aspect of SE, a lack of conceptual justification of indicator choice, a lack of measurement validation, undue length and unsuitability for monitoring in the general population [21]. The SEI-HS measures SE as a multidimensional concept involving cumulative disadvantages in the social, economic, cultural and political domains. It is based on extensive theoretical and empirical research [23, 24] and has been validated for the general population, as well as for the major non-Western immigrant groups in the Netherlands [25].

The aim of this study was to compare SE, as measured with the SEI-HS, with traditional social stratifiers as identifiers for high-risk/high-need population segments. We explored SE as a stratifier for health and low agency that potentially captures the information of most of the known stratifiers in a single measure. Our hypotheses were as follows:

1. SE is a stronger social stratifier than the commonly used social factors of education, income, labour market position and migration background.
2. SE is more strongly associated with low agency than the four abovementioned social factors.
3. Combining SE with one of the social factors will not improve its stratifying ability (as SE is the stronger social stratifier).

A social stratifier is considered to be stronger if it identifies strata with a larger health divide. The relative size of the health divide is measured by the relative risk (RR), and the absolute size is measured by the population attributable fraction (PAF). In epidemiology, the RR is the ratio of two risk estimates, and it is a statistic of choice for the comparison of risks between groups, as it is intuitively meaningful [26].^1^ The PAF estimates the proportion of the health problem that can be attributed to, or that is associated with, a particular risk factor, and thus represents the maximum health effect that can be achieved if the risk factor could be eliminated. We compared the RR and PAF of the stratifiers to identify at-risk populations for cardiovascular risk (diabetes, high blood pressure, smoking, obesity and inactivity), cancer, low self-rated health, anxiety and depression symptoms and low personal control, and we explored whether SE captures in a single measure the information that is normally obtained by the four abovementioned social factors. Data from the 2016 Public Health Monitor from four major cities in the Netherlands were used to test the hypotheses.

## Methods

### Data collection

The data in this study were collected by the Public Health Services of Amsterdam, Rotterdam, The Hague and Utrecht as part of the Public Health Monitor questionnaire 2016. The population sizes of the four cities ranged from 835 thousand in Amsterdam to 630 thousand, 520 thousand and 340 thousand in Rotterdam, The Hague and Utrecht, respectively. In each city, a stratified sample was drawn from the adult population aged ≥19 in non- institutionalised households based on neighbourhood and age category. Subjects were sent an invitation letter and up to three reminders by mail. The average response rate to the survey was 33.2%. Statistics Netherlands (CBS) enriched the monitoring data with administrative information regarding migration background, standardised household income and household composition.

### Measures

#### Dependent variables

- Health. The following measures were included:
  - ◦ Cardiovascular disease (CVD) risk factors: self-reported general practitioner (GP) diagnosis of diabetes; self-reported GP diagnosis of high blood pressure; current smoking; obesity (BMI 30 or higher based on self-reported height and weight); and inactivity (not meeting the daily recommended 30 min of moderate intensive physical activity on any day of the week).
  - ◦ Cancer: self-reported GP diagnoses of cancer;
  - ◦ Anxiety and/or depression symptoms: score 30 or higher on the 10-item Kessler Psychological Distress Scale (K10) versus score < 30 [27];
  - ◦ Self-rated health: fair or poor versus good or very good.
- Agency. The 7-item Pearlin Mastery Scale was used to measure the extent to which an individual regards his or her life chances as being under his or her personal control rather than fatalistically ruled: low (< 20) versus high (> = 20) personal control.

#### Independent variables

- Social exclusion. Social exclusion (SE) was measured with the Social Exclusion Index for Health Surveys (SEI-HS) [22]. The index consists of 17 items that measure four dimensions of SE: 1) social (limited social participation), 2) economic (material deprivation), 3) political (inadequate access to basic social rights) and 4) cultural (lack of normative integration). The scores were dichotomised into 1) moderate to strong exclusion and 2) some or no exclusion. As the cut-off point, we used the 95th percentile score in the 2012 Dutch adult population [22].
- Social stratifiers. The following four social factors were included:
  - ◦ Educational level: highest completed education (self-report) low, i.e., no schooling or elementary schooling versus low-middle, middle and high schooling;
  - ◦ Household income: standardised disposable annual household income after payment of income tax and social contributions lower than or equal to €16,100 versus higher^2^;
  - ◦ Labour market position: self-reported status low, i.e., unemployed, disabled for work and/or on social assistance, versus “other”, i.e., paid labour, retired, housewife/man and/or student; and
  - ◦ Migration background: mother and/or father born in a non-Western country versus born in the Netherlands and/or other Western countries (source: Statistics Netherlands).
- General characteristics. The following measures were included: sex, age and household composition. Age was treated as a continuous variable. Household composition was divided into four categories: family with children (i.e., living with partner, parent(s) and/or other adult(s) with children), family without children (ditto without children), single parent family and living alone.

### Statistics

First, we described the demographic composition, health, level of personal control and social stratification of the study population. To account for the complex sampling design and selective non-response, sample weights were calculated by Statistics Netherlands based on a linear model with 9 sociodemographic variables and their interaction terms [28]. For each of the measures, weighted descriptive statistics (percentages or means with standard deviations) were computed, and for the demographic measures, unweighted statistics were also computed.

Second, we estimated the relative risk with a 95% confidence interval (CI) for each health indicator and personal control by SE, education, income, labour market position and migration background. Complex samples cross tabs with relative risk tests were used. Herein, the RR represents the probability of a health indicator or personal control being present in the exposed group (P_SE+_ or P_SF+_) divided by the probability in the non-exposed group (P_SE_- or P_SF_-), in which SE is social exclusion and SF is one of the social factors; + is present and – is not present. An RR between 3 and 8 was considered strong, that between 1.8 and 3.0 was considered moderate and that between 1.4 and 1.8 was considered modest [26].

Third, we calculated the population attributable fractions from the RRs and the prevalences of SE and social factors (SF) with the following formulas:

$$ {\mathrm{P}\mathrm{AF}}_{\mathrm{SE}}={\mathrm{P}}_{\mathrm{SE}+}\ast \left({\mathrm{RR}}_{\mathrm{SE}}-1\right)/\left({\mathrm{P}}_{\mathrm{SE}+}\ast \left({\mathrm{RR}}_{\mathrm{SE}}-1\right)+1\right). $$

$$ {\mathrm{P}\mathrm{AF}}_{\mathrm{SF}}={\mathrm{P}}_{\mathrm{SF}+}\ast \left({\mathrm{RR}}_{\mathrm{SF}}-1\right)/\left({\mathrm{P}}_{\mathrm{SF}+}\ast \left({\mathrm{RR}}_{\mathrm{SF}}-1\right)+1\right). $$

PAFs and RRs were also calculated for the four dimensions of SE: limited social participation, material deprivation, inadequate access to basic social rights and lack of normative integration.

Finally, we calculated the overlap between SE and the four social factors, explored the contribution of SE to the stratifying power of the social factors and investigated the added value of combining SE with one of the social factors in terms of higher RRs and PAFs. Significance was assumed if there was no overlap between the 95% CIs.

## Results

### General characteristics

The sample consisted of 18,401 women (55.3%) and 14,884 men (44.7%), with a mean age of 57.1 years (SD 17.7) (Table 1). Almost half of the respondents lived with a partner and no children (46.4%), and over one-third (34.5%) lived alone. In the weighted sample, the mean age was lower, i.e., 44.9 years (SD 17.5), and the proportion living with partners and no children was lower (42.9%), as was the proportion living alone (22.7%). Weighted data were used in all subsequent analyses.

**Table 1:** General characteristics of the study sample, Public Health Monitor 2016 (*N* = 33,285)

	% / mean (SD)	
	Unweighted	Weighted ^a^
Demographics		
Female (%)	55.3	51.1
Mean age (sd)	57.1 (17.7)	44.9 (17.5)
Household composition (%)		
Family with children ^b^	13.8	21.7
Family without children ^c^	46.4	42.9
Single parent	5.3	7.4
Living alone	34.5	22.7

^a^ Sampling weights were calculated by Statistics Netherlands based on a linear model with 9 sociodemographic variables and their interaction terms [28]

^b^ Living with partner, parent(s) and/or other adult(s) with children; ^c^ ditto, without children

### Prevalence of health indicators, personal control, SE and social factors

Smoking was the most prevalent CVD risk factor, as one in four adults reported smoking (25.6%). Self-rated fair or poor health (SRH) was reported by 27.3%. We found a low score for personal control in 11.9% of the adult population and a low score for anxiety and depression symptoms in 9.4% of the population. (Table 2).

**Table 2:** Prevalence of health indicators and personal control (weighted ^a^)

	%
CVD risk factors	
Diabetes	7.0
High blood pressure	14.1
Current smoking	25.6
Obesity (BMI 30 or higher)	13.4
Inactivity	9.3
Cancer	2.8
Self-rated health fair or poor	27.3
Anxiety and depression symptoms	9.4
Low personal control	11.9

^a^ Sampling weights were calculated by Statistics Netherlands based on a linear model with 9 sociodemographic variables and their interaction terms [28]

One in ten adults were moderately to strongly socially excluded (10.3%); 9.0% reported a low educational level; 14.1% reported being unemployed, disabled for work, living on social assistance and without a paid job, and 31.8% of the adult population had a non-Western migration background. In the cities of Rotterdam and The Hague, these percentages were generally higher than those in Amsterdam and Utrecht. Only income showed a different pattern, with the highest rates of low income being found in Amsterdam and Rotterdam (Table 3).

**Table 3:** Prevalence of social exclusion and other social factors by city (weighted percentages^a^)

	Amsterdam	Rotterdam	The Hague	Utrecht	TOTAL
Social Exclusion					
SEI-HS index					
Moderate to strong	8.1	12.0	13.8	7.1	10.3
Some or no	91.9	88.0	86.2	92.9	89.7
Social Risk Factors					
Educational level (self-reported) ^b^					
Low	7.4	11.9	9.5	6.9	9.0
Not low	92.6	88.1	90.5	93.1	91.0
Standardised annual household income^c^					
Low: < 16,100 euro	26.0	25.4	22.1	23.8	24.7
Not low	74.0	74.6	77.9	76.2	75.3
Labour market position					
Low: unemployed, disabled, on social assistance	12.8	16.7	15.5	10.3	14.1
Not low	87.2	83.3	84.5	89.7	85.9
Migration background					
Native Dutch	49.5	52.3	48.7	68.7	52.9
Western migration background	18.2	12.1	17.0	11.2	15.3
Non-Western migration background	32.2	35.6	34.3	20.1	31.8

^a^ Sampling weights were calculated by Statistics Netherlands based on a linear model with 9 sociodemographic variables and their interaction terms [28]

^b^ Low: no or elementary schooling (PO); Not low: general secondary education, primary vocational education (MAVO, LBO); higher secondary education, secondary vocational training (HAVO, VWO, MBO); higher professional education and university (HBO, WO)

^c^ For this question, multiple answers were possible. The answers were categorised hierarchically with” > 20 h/week paid labour” first, followed by” 1–20 h/week paid labour” and” retired”. Those who checked “I am unemployed/job-seeking”, “I am disabled for work” or “I am on social assistance” and did not check one of the former three categories were classified as” unemployed, disabled, on social assistance”. The remaining respondents who checked “I am housewife/man” or “I am studying” were classified as “housewife/man or student”. Those considered “unemployed, disabled, on social assistance” were subsequently classified as low, and the remaining categories were classified as not low

### Performance of SE as social stratifier

**Fig. 1:**
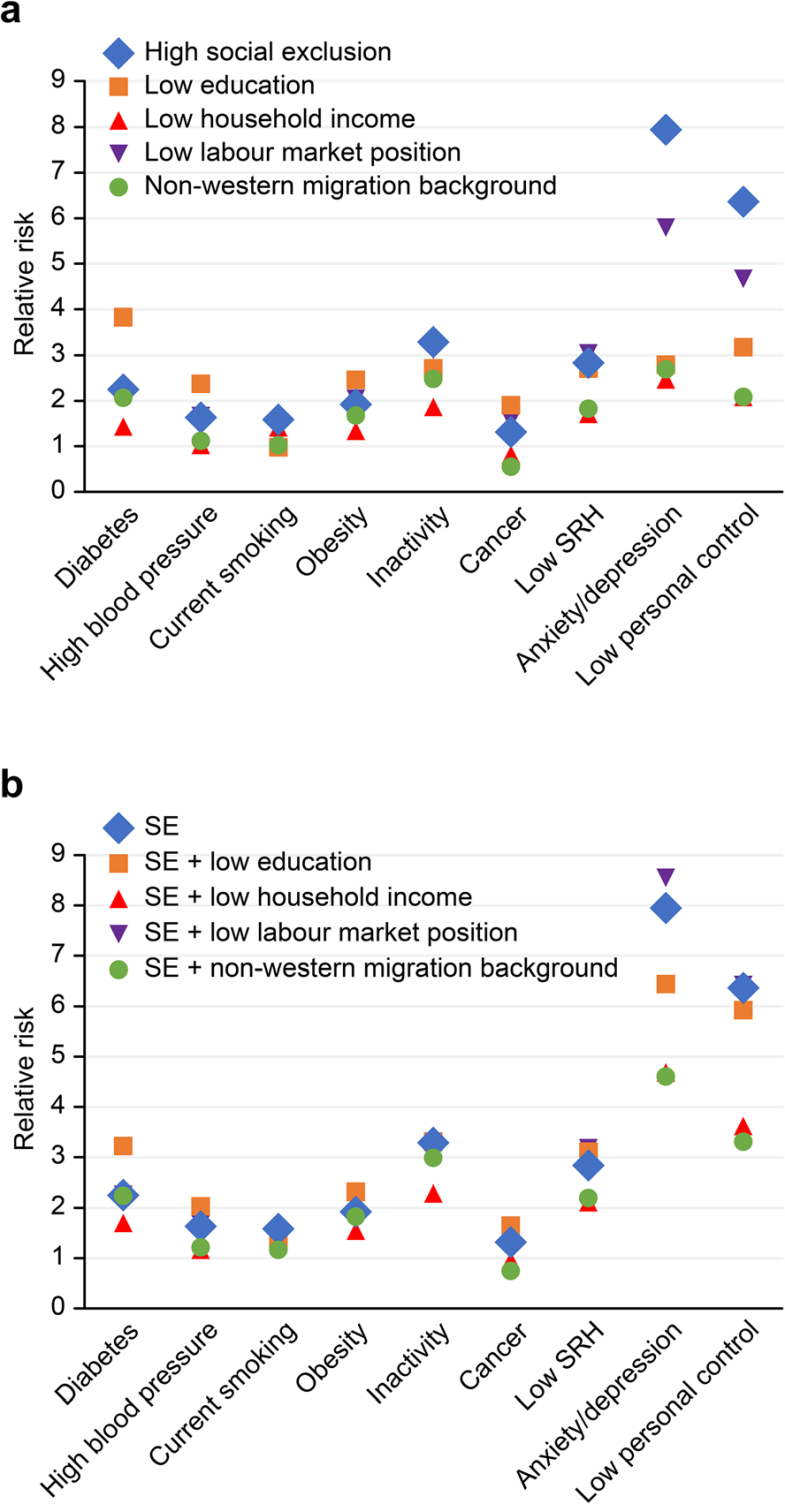
Relative risks of SE and four social factors, single (Panel **a**) and combined with SE (Panel **b**). Panel **a**. First orange dot: adults with low education had a 3.8 times higher risk of diabetes than other adults. Last orange dot: adults with low education had a 3.2 times higher risk of low personal control than other adults. Panel **b**. First orange dot: adults with low education and/or SE had a 3.2 higher risk of diabetes than other adults. Last orange dot: adults with low education and/or SE had a 5.9 times higher risk of low personal control than other adults

**Fig. 2:**
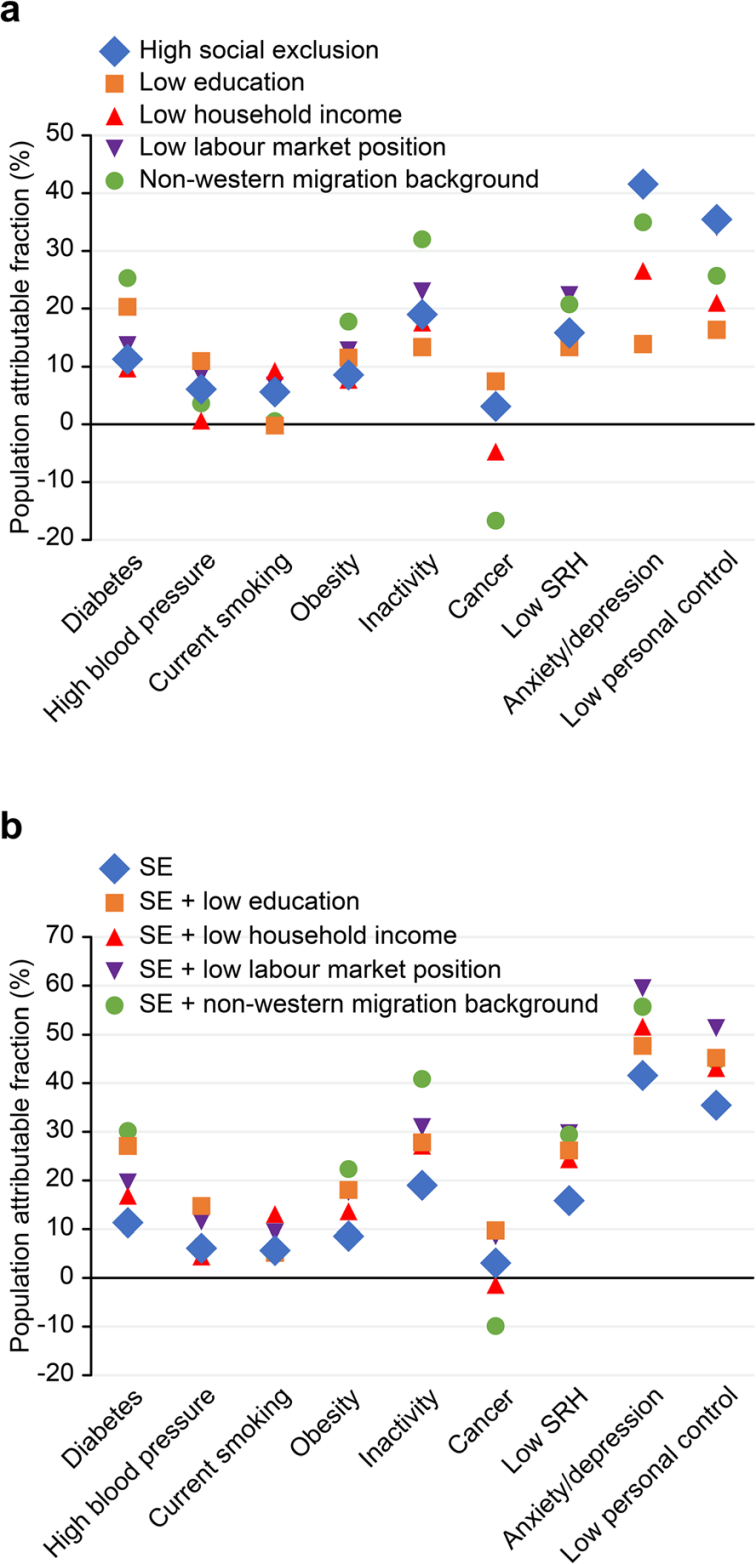
Population attributable fractions of SE and four social factors, single (Panel **a**) and combined with SE (Panel **b**). Panel **a**. First orange dot: if adults with low education would have the same risk of diabetes as other adults, the prevalence of diabetes would be reduced by 20%. Last orange dot: if adults with low education would have the same risk of low personal control as other adults, the prevalence of low personal control would be reduced by 27%. Panel **b**. First orange dot: if adults with low education and/or SE would have the same risk of diabetes as other adults, the prevalence of diabetes would be reduced with 16%. Last orange dot: if adults with low education and/or SE would have the same risk of low personal control as other adults, the prevalence of low personal control would be reduced by 45%

The RRs and PAFs for SE are listed in Table 4, columns 2 and 3, respectively. All relationships were significant at α = 0.05. The strength of the associations, however, varied considerably between health indicators. The RR was lowest for cancer (1.31) and highest for inactivity (3.29) and anxiety and depression symptoms (7.95). The PAF for cancer was 3.1%, and that for inactivity and anxiety and depression symptoms was 19.0 and 41.6%, respectively. The RR for low personal control was 6.36, with a PAF of 35.5%. This outcome signifies that socially excluded adults have a 6.36-fold higher chance of experiencing low personal control than non-excluded adults and that a hypothetical reduction of 35.5% in the prevalence of low personal control could be achieved if the socially excluded segment of the population were to have the same level of personal control as the rest of the population. An overview of RRs and PAFs is given in Figs. 1a and 2a.

**Table 4:** Relative risk (95% CI) and population attributable fraction for SE and social factors^b c^

	*Social exclusion (10.3%)*^*a*^		*Low education (9.0%)*^*a*^		*Low household income (24.7%)*^*a*^		*Low labour market position (14.1%)*^*a*^		*Non-Western migration background (31.8%)*^*a*^	
	RR	PAF	RR	PAF	RR	PAF	RR	PAF	RR	PAF
CVD risk factors										
♦ Diabetes	2.25 (1.96–2.57)	11.33	**3.83** ↑(3.42–4.28)	20.27	1.43 ↓(1.27–1.61)	9.66	2.12 (1.86–2.42)	13.65	2.06 (1.87–2.28)	25.30
♦ High blood pressure	1.63 (1.47–1.81)	6.09	2.37 ↑(2.17–2.59)	10.96	*1.03 ↓(0.94–1.12)*	*0.64*	1.66 (1.51–1.83)	8.53	1.12 ↓(1.03–1.21)	3.63
♦ Current smoking	1.58 (1.46—1.71)	5.64	*0.98 ↓(0.88–1.08)*	*−0.22*	1.41 (1.32–1.51)	9.27	1.51 (1.40–1.63)	6.73	*1.02 ↓(0.95–1.09)*	*0.57*
♦ Obesity	1.92 (1.72–2.14)	8.60	2.45 ↑(2.22–2.71)	11.57	1.34 ↓(1.22–1.47)	7.71	2.05 (1.85–2.26)	12.85	1.68 (1.54–1.82)	17.72
♦ Inactivity	**3.29** (2.92–3.70)	18.99	2.71 (2.40–3.06)	13.33	1.86 ↓(1.66–2.08)	17.54	**3.11** (2.78–3.50)	22.97	2.48 ↓(2.24–2.74)	32.01
Cancer	1.31 (1.02–1.69)	3.11	1.90 (1.53–2.36)	7.46	*0.82* ↓*(0.66–1.02)*	*−4.75*	1.56 (1.24–1.96)	7.30	0.55 ↓(0.44–0.69)	−16.68
Low self-rated health	2.83 (2.69—2.99)	15.83	2.71 (2.57–2.85)	13.33	1.71 ↓(1.62–1.80)	14.87	**3.04** (2.89–3.19)	22.31	1.82 ↓(1.73–1.92)	20.73
Anxiety /depression	**7.95** (7.19–8.78)	41.60	2.79 ↓(2.48–3.14)	13.87	2.46 ↓(2.21–2.75)	26.52	**5.79** ↓(5.22–6.43)	40.33	2.69 ↓(2.42–2.98)	34.93
Low personal control	**6.36** (5.87–6.91)	35.49	**3.17** ↓(2.88–3.50)	16.34	2.08 ↓(1.89–2.28)	20.98	**4.66** ↓(4.28–5.09)	34.06	2.08 ↓(1.91–2.28)	25.67

^a^ Weighted prevalences, population 19 years and older, G4, 2016

^b^ In *italic* if not significant at α = 0.05 and bold if RR strong, i.e., between 3 and 8 [26]

^c^ ↓ RR is significantly lower than the RR of SE alone, i.e., no overlap of 95% CIs, ↑ RR is significantly higher than the RR of SE alone

### Performance of other social factors

Table 4 columns 4 to 11 present the RRs and PAFs for each combination of social factors and health indicators. Low educational level showed a strong RR for diabetes (3.83) and moderate RRs for all other health indicators except current smoking, which was not significant. Low labour market position showed strong RRs for inactivity (3.11), low SRH (3.04) and anxiety and depression symptoms (5.79) and moderate RRs for diabetes and obesity. We did not find strong RRs in relation to low household income and non-Western migration background. Moderate RRs were found for inactivity and anxiety and depression symptoms by household income and for diabetes, inactivity, low SRH and anxiety and depression symptoms by non-Western migration background. The RRs for low personal control were strong for low education (3.17) and low labour market position (4.66) and moderate for low income and non-Western migration background. The PAFs showed a similar pattern.

### SE compared with other social factors

Figure 1a confirms that SE had much higher RRs for anxiety and depression symptoms and low personal control than did the four social factors (see also Table 4). These higher RRs resulted in higher PAFs for anxiety and depression symptoms and low personal control (Fig. 2a). The RRs of SE were also higher than the RR for smoking by low education; the RRs for diabetes, high blood pressure, obesity, cancer and low SRH by low income; and the RRs for high blood pressure, smoking and cancer by non-Western migration background. The RRs of SE were lower than the RRs for diabetes and high blood pressure by low education. In all other cases, the RRs of SE were not significantly different from those of the other four stratifiers (Fig. 1a, Table 4).

### Dimensions of SE

The RRs of the four dimension scales of the SEI-HS were found to be significant at α = 0.05 for all health indicators and low personal control, with two exceptions. Only the RRs of the cultural and political dimensions (inadequate access to basic social rights and lack of normative integration) for cancer were not significant. The social and economic dimensions (limited social participation and material deprivation) tended to show somewhat higher RRs than those for the political and cultural dimensions, especially for anxiety and depression symptoms and low personal control. The RRs and PAFs are shown in Table A1 (Additional file 1).

### Overlap SE and social factors and combined effect

To test the third hypothesis, we examined the overlap between the social factors and SE and the added value of combining SE with one of the social factors. Over one-third of adults with a low labour market position were socially excluded (34.1%). Moderate to strong social exclusion was also found in at least one in five adults with low education (25.7%), low household income (21.5%) or non-Western migration background (20.7%). Therefore, the overlap with SE was considerable, yet the social factors identified mainly non-excluded population groups (66–79%) (Table A2, Additional file 1).

**Fig. 3:**
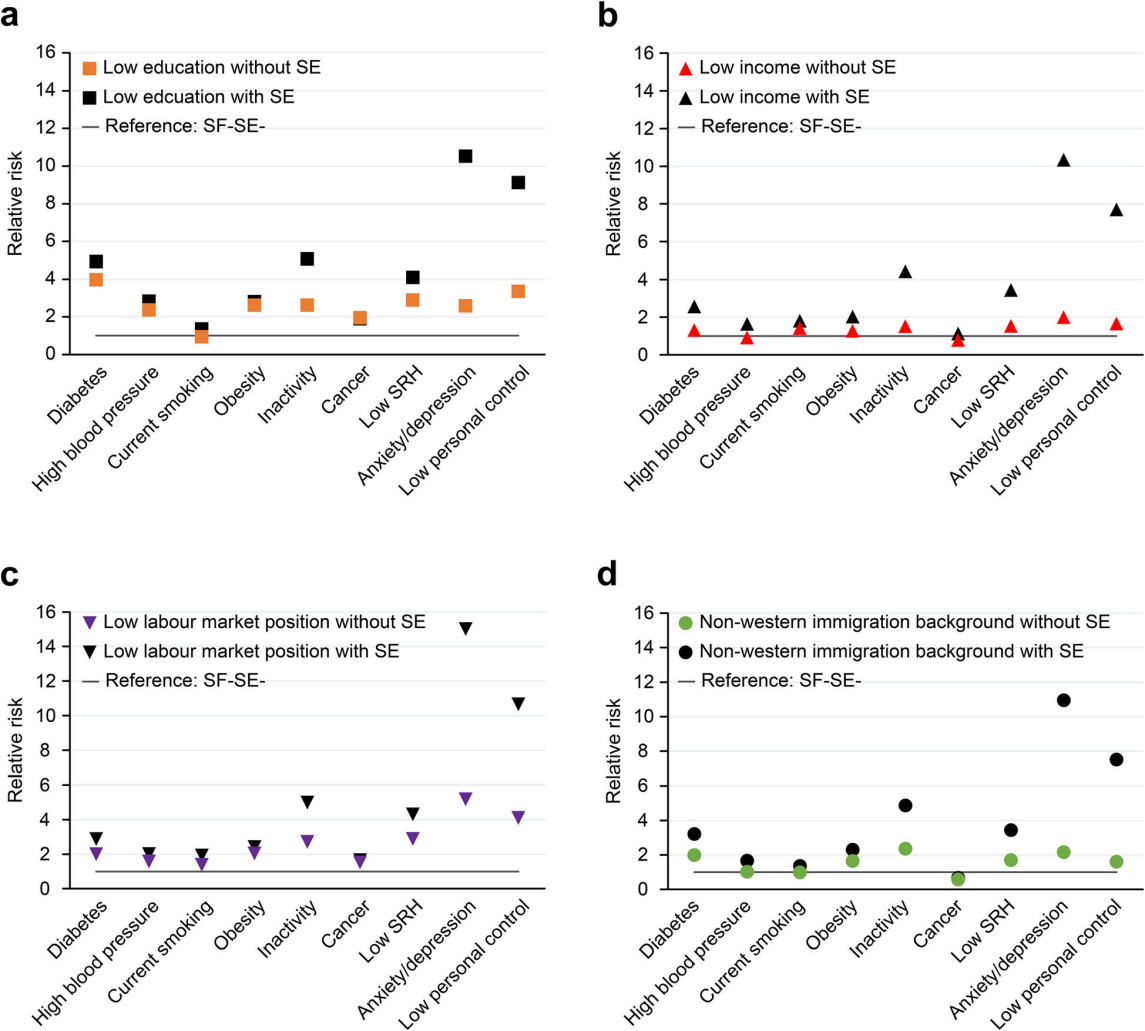
Relative risk by social factor SF, without and with SE, compared to the non-exposed group (SF-SE- group)

Figure 3 (and Table A3 Additional file 1) shows that for many health indicators, the RRs were lower in the non-excluded group than in the excluded group. The reference category here consisted of those who were not socially excluded and had no SF present (SE-SF- group). The reference value was set to 1. Figure 3a-d show that, especially for anxiety and depression symptoms and low personal control, the differences between the RRs were high. Respondents with low education and SE had an RR of 10.53 for anxiety and depression symptoms, while respondents with low education who were not socially excluded had an RR of 2.58, all of whom were compared to the non-exposed group (SE-SF- group) (Fig. 3a). For low labour market positions, the RRs of anxiety and depression symptoms were 15.02 when combined with SE and 5.17 when not (Fig. 3c). A large part of the stratifying power of low education and low labour market position is thus associated with SE. The same pattern can be seen for other health indicators and social factors, with a few exceptions; the ∆RRs of cancer, obesity and high blood pressure by low education and low labour market position and the ∆RR of diabetes by low education were not significantly higher with SE than without SE (Table A3 Additional file 1). In all other combinations, the RRs were significantly higher for the SF + SE+ group than for the SF + SE-group. It should be noted, however, that although the RRs in the SF + SE- group were generally lower, most of the RRs were significantly higher than 1 (31 out of 36) and would in other studies, with less pronounced results, be seen as relevant (Table A3 Additional file 1).

As shown in Table 5 and Figs. 1b and 2b, we investigated the potential contribution of the social factors to the stratifying power of SE. The panels show for each combination of SE and the social factors the RRs (1b) and PAFs (2b) for ill health and low personal control. The blue diamonds represent the RRs and PAFs of SE alone. In only three cases did the combination of SE with one of the social factors yield a higher RR than that of SE alone. The RRs for diabetes and high blood pressure increased when SE was combined with low education. This was to be expected, as we saw in Fig. 1a that the RRs of low education were significantly higher for diabetes and high blood pressure than the RRs of SE alone. Additionally, the RR for poor SRH increased when SE was combined with a low labour market position (Fig. 2a). In all other cases, combining SE with one of the four social factors resulted in equal or lower RRs (Table 5).

**Table 5:** Relative risk (95% CI) and population attributable fraction for combinations of SE and social factors^b c^

	*SE (10.3%)*^*a*^		*SE + low education (16.7%)*^*a*^		*SE + low household income (29.0%)*^*a*^		*SE + low labour market position (19.4%)*^*a*^		*SE + non-Western migration background (34.8%)*^*a*^	
	RR	PAF	RR	PAF	RR	PAF	RR	PAF	RR	PAF
CVD risk factors										
♦ Diabetes	2.25 (1.96–2.57)	11.33	**3.22** ↑(2.89–3.58)	27.07	1.70 ↓(1.52–1.89)	16.83	2.25 (2.01–2.53)	19.58	2.24 (2.03–2.47)	30.14
♦ High blood pressure	1.63 (1.47–1.81)	6.09	2.03 ↑(1.87–2.20)	14.69	1.16 ↓(1.07–1.26)	4.41	1.67 (1.53–1.82)	11.49	1.21 ↓(1.12–1.30)	6.76
♦ Current smoking	1.58 (1.46–1.71)	5.64	1.32 ↓(1.23–1.42)	5.15	1.52 (1.42–1.61)	13.01	1.53 (1.43–1.63)	9.31	1.16 ↓(1.09–1.24)	5.28
♦ Obesity	1.92 (1.72–2.14)	8.60	2.31 (2.12–2.52)	18.00	1.54 ↓(1.41–1.68)	13.60	2.10 (1.92–2.29)	17.57	1.82 (1.68–1.98)	22.29
♦ Inactivity	**3.29** (2.92–3.70)	18.99	**3.30** (2.96–3.67)	27.80	2.28 ↓(2.05–2.54)	27.11	**3.30** (2.96–3.68)	30.93	2.98 (2.69–3.30)	40.83
Cancer	1.31 (1.02–1.69)	3.11	1.64 (1.36–1.98)	9.74	*0.95 (0.79–1.15)*	−1.41	1.48 (1.22–1.81)	8.60	0.75 ↓(0.61–0.90)	−9.92
Low self-rated health	2.83 (2.69–2.99)	15.83	**3.11** (2.96–3.27)	26.14	2.11 ↓(2.00–2.22)	24.30	**3.18** ↑(3.02–3.34)	29.71	2.20 ↓(2.08–2.31)	29.40
Anxiety /depression	**7.95** (7.19–8.78)	41.60	**6.44** ↓(5.78–7.18)	47.67	**4.68** ↓(4.17–5.25)	51.63	**8.54** (7.63–9.57)	59.45	**4.60** ↓(4.10–5.17)	55.66
Low personal control	**6.36** (5.87–6.91)	35.49	**5.92** (5.44–6.45)	45.20	**3.61** ↓(3.31–3.95)	43.09	**6.42** (5.89–6.99)	51.30	**3.31** ↓(3.03–3.61)	44.56

^a^ Weighted prevalences, population 19 years and older, G4, 2016

^b^ In *italic* if not significant at α = 0.05 and bold if RR strong, i.e., between 3 and 8 [26]

^c^ ↓ RR is significantly lower than the RR of SE alone, i.e., no overlap of 95% CIs, ↑ RR is significantly higher than the RR of SE alone

The PAFs were all substantially higher than those for SE alone, as shown in Fig. 2b. For example, the RR and PAF for anxiety and depression symptoms by SE combined with low labour market position were 8.54 and 59.45, respectively, while those by SE alone were 7.95 and 41.60, respectively. This combination appears to be the most promising combination for population segmentation. Together, these two stratifiers, SE and low labour market position, identified 67.2% of the prevalence of anxiety and depression symptoms and 60.4% of the prevalence of low personal control in the adult population of the four study cities (Table 6). Worth mentioning are also the PAFs of inactivity (30.93), low SRH (29.71), diabetes (19.58), obesity (17.57) and high blood pressure (11.49) in this population segment (Table 5).

**Table 6:** Prevalence and proportion of ill health and personal control by population segment (%‘s, weighted ^a^)

	SE segment		SE + low labour market segment		
	Prevalence	Proportion^b^	Prevalence		Proportion^b^
CVD risk factors					
Diabetes	13.8	20.2	12.3		35.0
High blood pressure	21.4	15.5	20.3		28.4
Current smoking	38.2	15.3	35.5		26.9
Obesity (BMI 30 or higher)	23.5	17.6	23.0		33.0
Inactivity	24.3	26.6	20.3		43.8
Cancer	3.6	12.8	3.7		26.1
Self-rated health fair or poor	64.4	24.4	59.3		43.3
Anxiety and depression symptoms	42.4	47.5	31.5		67.2
Low personal control	48.6	41.7	35.5		60.4

^a^ Sampling weights were calculated by Statistics Netherlands based on a linear model with 9 sociodemographic variables and their interaction terms [28]

^b^ The proportion of the population with the condition in question, that falls within this segment. For example row 1 Diabetes: of the 174,134 socially excluded adults 24,030 or 13.8% reported diabetes. The socially excluded population segment thus accounted for 20.2% of all 118,965 diabetes cases (24,030/118,965)

## Discussion

Our first hypothesis, i.e., that SE is a stronger social stratifier than the commonly used social factors of education, income, labour market position and migration background, was confirmed for all four stratifiers in relation to anxiety and depression symptoms and for low household income and non-Western migration background in relation to the other health indicators. The second hypothesis, i.e., that SE is more strongly associated with low agency than the four social factors was also confirmed. The differences found for low personal control (as an indicator of low agency) were substantial. The third hypothesis, i.e., that combining SE with one of the social factors would not improve its stratifying ability, was confirmed in terms of RRs but not in terms of PAFs.

The study showed a remarkable 7.9-fold higher chance of experiencing anxiety and depression symptoms in socially excluded persons in urban areas of the Netherlands compared to individuals who were not socially excluded, which was significantly higher than that found for low education, low income, low labour market position and non-Western migration background. One might suspect overlapping symptoms between SE and anxiety and depression symptoms, but this was not found to be the case. SE and anxiety and depression are theoretically distinct concepts. Both were measured with validated instruments, namely, the SEI-HS for SE [22] and the Kessler-10 scale for anxiety and depression symptoms [27], respectively. The items of the scales reflect the different underlying concepts. The SEI-HS items ask, for example, about having enough money to heat one’s home, missing the pleasure of the company of others, satisfaction with one’s housing, giving money to good causes, etc., while the K10 scale items specifically ask about feeling tired, hopeless, restless, depressed, nervous, worthless, etc.. There are no overlapping items.

The K10 scale was originally developed to measure psychological distress, which is a common underlying factor in severe mental illness, in the general population [27] and has since been used to screen for anxiety and, in particular, depression [29, 30]. A high score on the K10 scale may indicate the presence of an anxiety or a depressive disorder, as well as a response to a specific stressor or demand [31]. Persons in a situation of social exclusion are, by definition, facing multiple problems in different domains of life, including economic and social domains and the lack of access to basic social rights. The emotional, cognitive and psychophysiological manifestations measured with the K10 scale may thus be a reaction to the situation that socially excluded people are generally in [31], as well as the result of prolonged exposure to chronic stressors in the form of depression, generalised anxiety and other psychological disorders [32, 33]. This may explain some of the associations found in this study.

In addition to differential exposure to stress, differences in coping mechanisms and resources may also influence the risk of psychosocial distress. SE citizens are exposed to more stressors, such as financial debts, loneliness, poor housing conditions and other social problems, and their coping mechanisms are also less effective than those of their counterparts. That is why the confirmation of the second hypothesis is crucial. People with a higher level of personal control may appraise themselves as being capable of coping with or controlling problems in their life and therefore may be less physiologically impacted by stressful events and ongoing situations [34, 35]. As they are more likely to view their health as controllable, they might exercise healthier behaviour and the better management of their health [35]. As almost 50% of the socially excluded citizens in the four Dutch cities reported low personal control, compared to 5.2% in the rest of the city population, this finding has implications for health care practice, public health interventions and social care in these cities.

Regarding physical disorders (diabetes, high blood pressure, obesity), lifestyle factors (smoking, inactivity) and low SRH, the first hypothesis was confirmed for low household income and non-Western migration background but not for low education and low labour market position. Low education (no or elementary schooling) and low labour market position (unemployed, disabled for work and/or on social assistance) appeared to be stronger social stratifiers in this population than were low income (lowest quintile disposable household income) and non-Western migration background. In the Netherlands, educational level is commonly used as the standard indicator of socioeconomic status in health research [36]. Our analyses showed that neither the less educated nor the other three social groups are homogeneous. We identified segments within these groups as those with higher and lower risks of ill health related to SE. Educational level, income and occupational status are good predictors of differences in (perceived) health but are not necessarily also the explanatory factors or the direction of solution [37]. Dutch health policies are now mainly aimed at compensating for a lack of knowledge through information, strengthening individual skills and promoting healthy behaviours, which is not enough to reduce health inequities [37].

The third hypothesis was confirmed only in terms of RRs. As SE is the strongest stratifier, combining SE with one of the four social factors did not lead to an increase in RRs. PAF is dependent not only on RR but also on the prevalence of exposure in the population. The proportion of people with SE and/or, for example, low education (16.7%) or low labour market position (19.4%) is of course higher than with SE alone (10.3%). The choice of whether to target a small group with a high RR or a larger population segment with a lower RR will depend on policy goals, opportunities and political values [38]. In-depth analyses per city can provide guidance here. From a population health perspective, one should consider the potential impact on those with different levels of risk for disease within a population, including those in underrepresented or underserved groups [39].

### Implications for policy, practice and research

We see a number of ways in which health care practice, public health interventions and social care services could be adapted to realise health gains for this population segment based on disease patterns and characteristics that influence the interaction with health and care services.

The first direction is taking agency into account in health care, public health and social care. In health care, a tailor-made and pro-active approach informed by data [40] could make a difference for persons with low agency, as could patient-centred care [41]. A promising development is DIABLEND, which is an integrated approach utilised in two deprived neighbourhoods in The Hague for personalised lifestyle optimisation in people with type-2 diabetes [42]. In public health, the focus for this group should be on the development and implementation of interventions that require little agency and explicitly enhance self-esteem and effective coping mechanisms [17, 43, 44] and increase social support as an important contributor to feelings of personal autonomy [45]. Good examples here are the Amsterdam Healthy Weight Programme that promotes a healthy food environment in which the healthy choice becomes the easy choice [46] and the municipality of Utrecht facilitating local support groups working together on building self-confidence, self-determination, healthy social relationships, meaningful roles and skills [47]. In social care too, agency should not be taken for granted. Pathways to Empowerment (Krachtwerk) is a good example, of a programme that is successfully applied in social and women’s shelters in the G4. This programme aims to improve the quality of the daily lives of persons who experience loss of control in their lives by focusing on their strengths and stimulating their personal agency, participation in society, and self-direction in life [48].

A second direction is addressing the convergence of health and social problems in this population segment. Cross-domain working is still in its infancy and in practice, it is hard to get off the ground [3]. The Dutch programme” The Right Care in the Right Place” sets an example by advocating a different perspective on sickness and health, with more focus on what people need to be able to function and less on what the care system has to offer, starting with people’s capabilities, vitality, resilience and wishes [3]. A good example is the introduction of Powerful Basic Care (Krachtige Basiszorg) within deprived areas in the G4 [49]. In social care, more attention should be given to health and health promotion.

A third direction is paying more attention to upstream policies at the meso and macro levels. SE is not just an individual problem. Lack of social cohesion, discrimination and stigma, deprived neighbourhoods, complex bureaucratic procedures, individualization, high demands on people’s self-reliance and lagging social benefits are all factors that affect SE and health. The issue we should pursue is how to ensure that people who are on social benefits, and those we are unemployed or disabled and cannot work, can participate fully in our society; i.e., how do we make our institutions inclusive and build up self-respect and agency instead of distorting these capabilities? A good example here is the application of scientific evidence, e.g., on Mobility Mentoring®, to create stress-sensitive services within the municipality of Utrecht [47]. Room for future social experiments and comparative research is needed.

The fourth and last direction is not forgetting those who have already fallen through the cracks of society, i.e., the homeless, people living in protected and sheltered housing, detainees and undocumented immigrants, all of whom did not participate in this research. It is important to incorporate these groups in regular health care, prevention and social policies to prevent further exclusion.

### Strengths and limitations

This study has some major strengths and limitations. The strong points include the use of a large representative sample, the inclusion of all major lifestyle and health outcomes in terms of mortality and morbidity and the employment of validated instruments to measure social exclusion, anxiety and depression symptoms and personal control. The limitations are as follows. First, as in any cross-sectional study, no causal relations could be examined. The PAFs calculated in this study are largely theoretical and do not necessarily hold in practice. The PAFs herein represent the proportional reduction in overall morbidity or unhealthy behaviour that would occur if the lowest social stratum would experience the same rate as the rest of the population. No rigorous statistical testing took place, as this was not considered relevant for the purpose of the research and the exploratory nature of the study. In addition, confounding has not been taken into account. Our goal was to identify population segments with high levels of ill health and low personal control in a given context. In a different social context, a comparable study could lead to different results. We expect, based on additional analyses per city that are not shown herein but are available from the authors, that the results could be generalised to urban areas with similar socioeconomic characteristics. To allow for future generalizations, factors at the meso and macro levels should be included, such as urbanicity, neighbourhood characteristics, welfare and social policies. In this study, we treated SE, education, income, labour market position and migration background as micro-level characteristics of individuals, while these factors also reflect the underlying social and economic structure. Another limitation of this study is that persons without a fixed address and those living in institutions were not included in the Public Health Monitor, which could have led to an underestimation of the RRs and PAFs. A final limitation is that most health indicators were self-reported. Self-reported measures are prone to social desirability bias and recall bias. There are no concrete indications for differences between social groups in the magnitude or direction of these biases, but it cannot be ruled out.

## Conclusions

This study shows that the SEI-HS is a powerful tool for identifying high-risk/high-need population segments in which not only ill health is concentrated, as is the case with traditional social stratifiers, but also an extremely high prevalence of anxiety and depression symptoms and low personal control are present, in addition to an accumulation of multiple problems in different domains of life. The combination of SE with a low labour market position captured the largest part of the prevalence of anxiety and depression symptoms (67%) and low personal control (60%) in 19.5% of the population, as well as a substantial portion of other risk factors and negative health outcomes. Significant health gains are likely to be achieved by tailoring health care practice, public health interventions and social care to the needs and capacities of this socially excluded and low labour market group. More in-depth analysis of PHM data is recommended at the local level to sharpen the local profile of the socially excluded population segments per city. In general, more qualitative research, comparative studies and experiments are needed regarding the impact and interaction of meso- and macro-level factors on the triangle formed by SE, health and low agency.

## Supplementary Information

**Additional file 1: Table A1.** Relative risk (95% CI) and PAF for four dimensions of social exclusion. **Table A2.** Overlap between social exclusion and four social factors (weighted percentages). **Table A3.** Relative risks (95% CI) for social factors with and without SE and differential effects.

## Data Availability

The dataset used and analysed in the current study is available from the GGD Rotterdam-Rijnmond (Gezondheidsmonitor@rotterdam.nl) upon reasonable request.

## Abbreviations

BMI: Body mass index
CI: Confidence interval
CVD: Cardiovascular disease
GP: General practitioner
K10: 10-item Kessler psychological distress scale
PAF: Population attributable fraction
PROGRESS: Place of residence, race or ethnicity, occupation, gender, religion, education, socioeconomic status and social capital or resources
RR: Relative risk
SD: Standard deviation
SE: Social exclusion
SEI-HS: Social Exclusion Index for Health Surveys
SF: Social factor
SRH: Self-rated health
WHO: World Health Organization

## Footnotes

1 While the frequently used odds ratio (OR) is an algebraic transformation of probabilities, the relative risk is intuitively more meaningful. To give an example: suppose that 30% of men and 10% of women in a given population have diabetes. The OR of men compared to women in this population is 3.9, which is calculated as (0.3/0.7)/(0.1/0.9). The RR of men compared to women is 3.0, which means that diabetes is 3-times as common in men than in women. The latter result is easy to grasp, while the first is quite abstract and difficult to explain to policy makers and practitioners. In practice, the OR is often interpreted as a RR. This is acceptable when the outcome is rare (< 10%) as the value of OR will not be too different from that of RR. However, as the prevalence increases, the two ratios diverge, and the OR will tend to exaggerate the strength of the association [26]. Hence, we have a preference for the use of RRs in this study.

2 **€**16,100 corresponds to the lowest income quintile in the Netherlands **(**source: Statistics Netherlands)**.**

## Glossary

Social exclusion: - The cumulation of disadvantages in social, economic, cultural and political domains. A person is socially excluded if he/she cannot participate fully in society and make use of the benefits that society offers.
Social Exclusion Index for Health Surveys (SEI-HS): - Validated instrument to measure the multidimensional concept of social exclusion.
Agency: - The human capability to influence one’s functioning and the course of events by one’s actions.
Personal control: - The extent to which an individual regards his or her life chances as being under his or her personal control rather than fatalistically ruled.
Relative risk (RR): - The risk of a certain event (disease, risk factor, etc.) in one group compared to the risk of the same event in another group.
Population attributable fraction (PAF): - The proportion of the health problem that can be attributed to, or that is associated with, a particular risk factor.
