# Supplementary material for: The cumulation of ill health and low agency in socially excluded city dwellers in the Netherlands: how to better identify high-risk/high-need population segments with public health survey data
Source: Int J Equity Health. 2021 Jul 19;20:167. doi: 10.1186/s12939-021-01471-w (PMC8290568; doi:10.1186/s12939-021-01471-w)
Supplement: Supplementary file 1 — Additional file 1: Table A1. Relative risk (95% CI) and PAF for four dimensions of social exclusion. Table A2. Overlap between social exclusion and four social factors (weighted percentages). Table A3. Relative risks (95% CI) for social factors with and without SE and differential effects. [file 12939_2021_1471_MOESM1_ESM.pdf]

Table A1. Relative risk (95% CI) and PAF for four dimensions of social exclusion

	*Dimension 1: Limited social participation (9.8%)**		*Dimension 2: Material deprivation (8.9%)**		*Dimension 3: Inadequate access to basic social rights (10.0%)**		*Dimension 4: Lack of normative integration (8.6%)**		*Social Exclusion Index (10.3%)**	
	RR	PAF	RR	PAF	RR	PAF	RR	PAF	RR	PAF
CVD and risk factors										
♦ CVD	2.18 (1.65-2.89)	11.55	**3.01** (2.28-3.99)	18.02	1.28 (0.93-1.75)	2.78	1.47 ((1.11-1.94)	4.00	2.58 (1.95-3.41)	13.93
♦ Diabetes	1.71 (1.46-1.99)	6.88	2.35 (2.05-2.70)	12.10	1.47 (1.26-1.73)	4.74	1.50 (1.28-1.75)	4.28	2.25 (1.96-2.57)	11.33
♦ High blood pressure	1.41 (1.26-1.58)	4.03	1.93 (1.74-2.15)	8.33	1.23 (1.09-1.39)	2.31	1.21 (1.07-1.36)	1.76	1.63 (1.47-1.81)	6.09
♦ Current smoking	1.35 (1.24-1.48)	3.43	1.57 (1.45-1.71)	5.12	1.44 (1.32-1.56)	4.36	1.39 (1.27-1.52)	3.38	1.58 (1.46-1.71)	5.64
♦ Obesity	1.53 (1.36-1.72)	5.13	2.16 (1.94-2.41)	10.39	1.57 (1.39-1.77)	5.67	1.46 (1.29-2.51)	3.97	1.92 (1.72-2.14)	8.60
♦ Inactivity	2.80 (2.46-3.18)	17.52	**3.03** (2.66-3.45)	18.19	2.02 (1.75-2.33)	10.19	2.20 (1.92-2.51)	10.32	**3.29** (1.92-3.70)	18.99
Cancer	1.51 (1.15-1.99)	4.97	1.35 (1.04-1.77)	3.14	*1.13 (0.87-1.46)*	1.28	*1.22 (0.90-1.65)*	1.86	1.31 (1.02-1.69)	3.11
Low self-rated health	2.41 (2.28-2.56)	13.79	2.84 (2.69-3.00)	16.44	2.10 (1.98-2.24)	11.04	1.59 (1.48-1.72)	5.10	2.83 (1.69-2.99)	15.83
Anxiety /depression	**5.38** (4.94-5.86)	42.72	**5.01** (4.59-5.48)	35.89	**3.31** (3.00-3.65)	23.10	2.33 (2.08-2.62)	11.46	**7.95** (7.19-8.78)	41.60
Low personal control	**6.70** (6.05-7.42)	55.62	**5.95** (5.36-6.60)	44.27	**3.81** (3.40-4.27)	28.09	2.77 (2.43-3.16)	15.21	**6.36** (5.87-6.91)	35.49

* Weighted prevalence, population 19 years and older, G4, 2016.

# In *italic* if RR <u>not</u> significant at $\alpha = 0.05$ and **bold** it RR strong, i.e., between 3 and 8 [26].

Table A2. Overlap between social exclusion and four social factors (weighted percentages).

	Low education			*Low household income*			*Low labour market position*			*Non-Western migration background*		
Social exclusion	No	Yes	Total	No	Yes	Total	No	Yes	Total	No	Yes	Total
No	83.3	6.6	89.8	71.0	19.0	90.0	80.6	9.3	89.8	65.2	24.6	89.7
Yes	7.9	2.3	10.2	4.8	5.2	10.0	5.4	4.8	10.2	3.8	6.4	10.3
Total	91.1	8.9	100.0	75.8	24.2	100.0	85.9	14.1	100.0	69.0	31.0	100.0
Proportion SE	8.7	25.7		6.4	21.5		6.2	34.1		5.6	20.7	

Table A3. Relative risks (95% CI) for social factors with and without SE and differential effects [# $].

	RR_{SF+SE+}	RR_{SF+SE-}	$\Delta(RR_{SF+SE+}, RR_{SF+SE-})$
**Low education**			
CVD risk factors			
♦ Diabetes	**4.93 (4.07-5.97)**	**3.97 (3.49-4.52)**	0.96 .
♦ High blood pressure	2.84 (2.45-3.30)	2.36 (2.13-2.62)	0.48 .
♦ Current smoking	1.35 (1.15-1.59)	*0.93 (0.81-1.06)*	0.42 ↓
♦ Obesity	2.82 (2.37-3.35)	2.62 (2.33-2.94)	0.20 .
♦ Inactivity	**5.07 (4.24-6.07)**	2.62 (2.26-3.04)	2.45 ↓
Cancer	1.89 (1.21-2.98)	1.96 (1.53-2.50)	-0.06 .
Low Self-Rated Health	**4.09 (3.82-4.39)**	2.89 (2.71-3.09)	1.20 ↓
Anxiety/depression symptoms	**10.53 (9.14-12.13)**	2.58 (2.16-3.08)	7.95 ↓
Low personal control	**9.13 (8.12-10.27)**	**3.35 (2.91-3.85)**	5.78 ↓
**Low household income**			
CVD risk factors			
♦ Diabetes	2.56 (2.13-3.08)	1.31 (1.13-1.51)	1.26 ↓
♦ High blood pressure	1.64 (1.42-1.90)	*0.91 (0.82-1.02)*	0.73 ↓
♦ Current smoking	1.80 (1.62-2.01)	1.41 (1.30-1.52)	0.40 ↓
♦ Obesity	2.03 (1.75-2.36)	1.27 (1.13-1.42)	0.76 ↓
♦ Inactivity	**4.43 (3.82-5.14)**	1.52 (1.31-1.76)	2.91 ↓
Cancer	*1.13 (0.77-1.65)*	*0.78 (0,60-1.01)*	0.35 .
Low Self-Rated Health	**3.45 (3.23-3.67)**	1.53 (1.42-1.64)	1.92 ↓
Anxiety/depression symptoms	**10.35 (9.10-11.76)**	1.99 (1.69-2.34)	8.36 ↓
Low personal control	**7.71 (6.95-8.54)**	1.66 (1.45-1.90)	6.05 ↓
**Low labour market position**			
CVD risk factors			
♦ Diabetes	2.87 (2.37-3.49)	1.99 (1.68-2.35)	0.89 ↓
♦ High blood pressure	2.01 (1.74-2.33)	1.57 (1.39-1.78)	0.44 .
♦ Current smoking	1.92 (1.73-2.12)	1.37 (1.25-1.51)	0.55 ↓
♦ Obesity	2.42 (2.08-2.81)	2.04 (1.80-2.29)	0.38 .
♦ Inactivity	**4.98 (4.29-5.79)**	2.71 (2.33-3.15)	2.28 ↓
Cancer	1.65 (1.13-2.42)	1.52 (1.16-2.01)	0.13 .
Low self-Rated Health	**4.30 (4.06-4.55)**	2.88 (2.70-3.08)	1.42 ↓
Anxiety/depression symptoms	**15.02 (13.29-16.97)**	**5.17 (4.42-6.06)**	9.84 ↓
Low personal control	**10.67 (9.69-11.74)**	**4.10 (3.60-4.66)**	6.57 ↓
**Non-Western migration background**			
CVD risk factors			
♦ Diabetes	**3.21 (2.72-3.80)**	1.99 (1.76-2.23)	1.23 ↓
♦ High blood pressure	1.67 (1.46-1.91)	*1.03 (0.93-1.13)*	0.64 ↓
♦ Current smoking	1.37 (1.23-1.54)	*0.99 (0.91-1.07)*	0.39 ↓
♦ Obesity	2.30 (1.97-2.65)	1.65 (1.49-1.82)	0.65 ↓
♦ Inactivity	**4.88 (4.21-5.65)**	2.36 (2.09-2.67)	2.52 ↓
Cancer	*0.68 (0.45-1.05)*	0.57 (0.44-0.74)	0.11 .
Low self-Rated Health	**3.44 (3.22-3.67)**	1.70 (1.59-1.82)	1.73 ↓
Anxiety/depression symptoms	**10.95 (9.64-12.44)**	2.16 (1.85-2.52)	8.79 ↓
Low personal control	**7.52 (6.79-8.32)**	1.60 (1.41-1.82)	5.91 ↓

In *italic* if RR not significant at $\alpha = 0.05$ and **bold** it RR strong i.e. between 3 and 8 [26].

& ↓ RR_{SF+SE-} is significantly lower than RR_{SF+SE+}, i.e., there is no overlap between the 95% CIs.
